# Supplementary material for: Are carbon emissions trading and green financial instruments synergistic? -Comprehensive quantitative research based on content analysis
Source: PLoS One. 2024 Mar 7;19(3):e0298601. doi: 10.1371/journal.pone.0298601 (PMC10919601; doi:10.1371/journal.pone.0298601)
Supplement: S1 Data — (ZIP) [file pone.0298601.s001.zip › Data/地级市Z_F工作报告.zip!/─┌├╔╣┼/│α╖σ╩╨/└┤╫╘╣1⁄2╓┌║┼í╛┬φ┐╦╩2╛▌═°í┐_2017.docx]

各位代表：

　　现在，我代表市人民政府，向大会报告工作，请予审议，并请市政协委员和其他列席的同志们提出意见。

　　2016年工作回顾

　　过去的一年，面对经济下行压力持续加大、重点工作任务复杂艰巨的严峻形势，市政府认真贯彻中央、自治区和市委的各项决策部署，紧紧依靠全市各族人民，戮力同心，攻坚克难，较好地完成了市六届人大四次会议确定的目标任务，实现了十三五良好开局。全市地区生产总值完成1933亿元，增长7.3%；一般公共预算收入111.8亿元，增长6.9%，按可比口径增长9.8%；500万元以上固定资产投资1469亿元，增长15.5%；社会消费品零售总额700亿元，增长9.9%；城镇和农村牧区常住居民人均可支配收入达到27336元和9517元，分别增长8.5%和8%。

　　一年来，我们主要做了以下工作：

　　一是以全面落实五大任务为主线，有效保证了经济稳定增长。突出抓好三去一降一补。全年淘汰钢铁落后产能31万吨、煤炭落后产能30万吨。持续实施去库存系列措施，房地产市场总体稳定，商品房销售面积增长10.7%，待售面积下降17.7%。开展了打击非法集资、金融诈骗等专项行动，有效防控了金融风险。加大实体经济扶持力度，全面推行营改增政策，实施企业登记注册五证合一、一照一码和个体工商户两证整合改革措施，累计为市场主体降低成本17亿元，新增市场主体5.1万户。实施了9大类45项补短板工程，基础设施、生态环境、科技创新等领域的主要问题得到有效改善。突出抓好项目建设。实施投资5000万元以上重点项目737个，完成投资961.4亿元，同比增长15.6%；实施招商引资和项目建设冬季攻坚行动，99个项目签约落地，374个项目前期加快推进，策划投资1000万元以上项目1200个。突出抓好经济运行调度。克服严重旱灾的不利影响，粮食总产保持在百亿斤以上，家畜存栏达到2231万头只、居自治区首位。积极稳定工业生产，推动81户停产的规模以上企业实现复产，规模以上工业增加值增长7%。全市金融机构存贷款余额分别达到1842.6亿元和1381.5亿元，分别增长23.3%和18.8%。

　　二是以培育壮大六大产业为抓手，有效推动了产业结构转型升级。现代农牧业：全面实施3661工程，取得了玉米种植面积、肉羊养殖规模两减和设施农业、人工种草、高效丰产经济林、生猪养殖、肉牛养殖五增的可喜成效，较好完成年度工作目标。荣获中国牛羊肉食品之都称号。冶金产业：云铜40万吨铜冶炼项目奠基开工，金剑40万吨铜冶炼项目签约落地，有色金属产业综合体及非标有色金属交易中心启动建设。清洁能源产业：落实了京津冀1000万千瓦的新能源电力市场消纳承诺，赤峰（元宝山）至华北500千伏输电通道列入国家十三五电力发展规划，国电投2×30万千瓦热电联产项目和26万千瓦新能源电力项目建成竣工。战略性新兴产业：新能源汽车、航空装备制造产业基地项目合作签约，智慧赤峰建设全面启动，蒙东云计算中心入驻企业达到86家。旅游业：与南开大学建立了旅游创新发展战略合作关系，阿斯哈图石林景区通过国家5A级景区资源评价，新增国家4A级旅游景区两家，全年接待游客1453万人次，旅游收入228亿元。宁城县和克旗进入国家全域旅游示范区创建行列。玉龙机场旅客吞吐量突破100万人次。商贸物流业：全市物流园区销售收入突破500亿元，红山物流园区获国家示范物流园区称号。左旗、林西县、克旗、翁旗、敖汉旗成为全国电子商务进农村示范县建设试点，红山区被纳入国家服务业综合改革试点地区。

　　三是以搭建四个平台为支撑，有效破解了瓶颈要素制约。壮大工业发展平台。编制完成赤峰城区东部工业经济带总体规划，出台了飞地经济政策，赤峰经济开发区晋升国家高新区完成申报工作。马林有色金属产业园区启动建设，2×35万千瓦自备电厂获批。创新投融资平台。39个PPP项目开工建设或落实社会资本方，撬动社会投资360亿元。市本级设立产业基金11支，引进金融资本233亿元，通过其他金融工具成功融资55.1亿元。新增4家企业在新三板挂牌、3家企业在区域性股权交易市场挂牌。整合组建了城投、交通、兴农、文旅四大投融资集团，资产总额达1758亿元，政府的投融资能力大幅提升。优化对外开放平台。举办了内蒙古牛羊肉产业大会、京津冀蒙旅游推介大会、蒙冀辽文化产业博览会，与广东省湛江市缔结友好城市。对外贸易回稳向好，开通运行赤满欧国际货运班列，保税物流中心进出口总额增长5.6倍，全市外贸进出口总额增长11.3%。全年引进市外资金659亿元，同比增长11.1%。完善科教人才支撑平台。成功举办了京蒙科技合作对接大会和中国北方农博会，开展了科技列车赤峰行系列活动，新增自治区级高新技术产业化基地1家。全市农村牧区中小学办学条件提升工程启动实施，开工率达87.9%。阿旗、右旗、元宝山区通过国家义务教育均衡发展验收，全市高考本科上线率提高5个百分点。完成职教园区选址，规划论证和社会资本方选定工作正在推进。

　　四是以打好五大攻坚战为重点，有效增强了发展保障能力。脱贫攻坚开局良好。投入资金84.3亿元，实现了5.76万贫困人口稳定脱贫，超额完成年度任务。十个全覆盖工程基本完成。三年累计投入资金512.6亿元，村庄覆盖率达77.4%，农牧民人口覆盖率达92%。新型城镇化加快推进。中心城区50公里地下综合管廊、50公里环城水系治理项目开工建设，总理关注的铁南棚改全面完成，公共自行车租赁系统、沿河自行车道、污泥处理系统投入运营，城市绿亮美工程一期完成建设。生态环保工作成效明显。完成营造林111.2万亩、水土保持综合治理151万亩，封育禁牧立法工作基本完成。实施重点区域绿化41万亩，栽植苗木4000万株，完成投资40亿元。扎实开展环境整治百日攻坚行动，有效排查治理环境突出问题426个，完成自治区下达的污染治理和四项主要污染物减排任务。重大基础设施建设进展顺利。启动实施千亿元工程包，当年完成投资210亿元。经棚至锡林浩特高速公路具备通车条件，高铁项目全线开工，跨区域调水工程顺利启动，朝阳至赤峰燃气管道开工建设，3个220千伏输变电扩建工程竣工投运。

　　五是以推进五大工程为依托，有效增进了民生福祉。全市民生支出346.3亿元，占财政支出比重78.8%。就业创业工程：新增国家级众创空间3家，通过政府购买服务引进了北京双创服务龙头企业，共同打造赤峰安快创业谷。城镇新增就业3.2万人，农牧民转移就业70.6万人，完成各类就业创业技能培训3.7万人次。社会保障工程：完成城乡医保整合工作，社会保险综合覆盖率稳定在90%以上。城乡低保标准分别提高492元和301元，为75.6万户低收入农牧户发放取暖用煤补贴，为低保家庭大学新生发放1.5亿元就学补助，零就业家庭保持动态清零。健康赤峰工程：赤峰学院附属医院晋升为三级甲等医院，县级公立医院综合改革全面深化，全科医生和乡村医生签约服务覆盖率达到85%。食品药品监管体制改革全面完成。文明创建工程：群众艺术馆和青少年宫建成并投入使用，话剧《热土》等文艺作品在区内外引起较大反响。荣获国家双拥模范城八连冠和全国人防先进城市四连冠。社会建设工程：持续开展安全生产大检查大督查及煤矿等重点领域专项整治，实施全市公路安全畅通工程，不断深化平安赤峰建设，获得全国法治宣传教育先进市和全国创新社会治理优秀城市荣誉称号。

　　六是以打造廉洁务实高效政府为目标，有效加强了自身建设。深入开展两学一做学习教育，扎实推进不作为、慢作为、乱作为专项整治。完成了公务用车制度改革，强化财政资金监管，通过财政投资评审、公共资源集中交易节约资金69.3亿元，全市三公经费支出下降2.6%。深化以放管服为重点的审批制度改革，向社会公布了市县权责清单和中介服务事项清单。加强行政监察和审计监督，有效规范了行政权力运行。自觉接受社会各界监督，主动向人大报告工作，制定了市政府重大事项向市人大及其常委会报告制度，加强与政协的沟通联系和民主协商，全年办理人大代表建议和政协委员提案293件。

　　社会科学、妇女儿童、残疾人、红十字、慈善等事业稳步发展，民族宗教、外事侨务、史志档案、防震减灾、气象服务、人防消防等工作进一步加强。

　　各位代表，过去一年，我们取得的成绩来之不易。这是自治区党委、政府和市委总揽全局、正确领导的结果，是市人大、市政协履职监督、鼎力支持的结果，是全市上下群策群力、务实拼搏的结果。在此，我代表市人民政府，向全市广大干部群众，向所有关心、支持、参与赤峰改革开放和现代化建设的同志们、朋友们表示衷心的感谢！

　　成绩固然值得肯定，困难和问题也不容忽视。主要是：经济总量小、产业层次低的问题没有根本改善，发展方式仍然粗放，结构调整空间有限。经济下行压力仍然较大，实体经济特别是工业企业困难较多，企业融资成本较高，民间投资增速回落明显。改善民生任务繁重，城乡居民增收困难，脱贫攻坚任务紧迫而艰巨，群众关心的上学、就医、养老、出行等热点难点问题仍然突出。生态环境总体脆弱，节能减排压力明显增大。社会矛盾易发多发，安全生产形势十分严峻，维护稳定工作面临很多新情况新挑战。政府职能转变还不到位，个别政府部门和工作人员的担当意识、服务意识不强，廉政建设和作风建设仍需加强。特别是宝马公司特大安全生产事故，给人民群众造成了严重的生命和财产损失，集中暴露了干部作风和政府工作存在不严不实的问题，教训极其惨痛。对于这些问题，我们一定要保持清醒认识，以担当进取的作风、革故鼎新的勇气，全力以赴加以解决。

　　2017年工作安排

　　今年是实施十三五规划承上启下的关键之年，我们还将迎来党的十九大胜利召开和自治区成立70周年，做好今年的工作意义重大、影响深远。今年政府工作的总体要求是：深入贯彻习近平总书记系列重要讲话精神和治国理政新理念新思想新战略，全面落实自治区第十次党代会和市第七次党代会精神，坚持稳中求进工作总基调，深入推进供给侧结构性改革，坚决守住发展、生态和民生三条底线，狠抓项目建设，突出产业发展，注重城乡统筹，持续改革开放，切实改善民生，确保安全稳定，奋力加快赤峰跨越崛起步伐，为全面建成小康社会奠定更加坚实的基础，以优异成绩庆祝自治区成立70周年，迎接党的十九大胜利召开。

　　今年经济社会发展主要预期目标是：地区生产总值增长8%左右，一般公共预算收入按可比口径增长8%，规模以上固定资产投资增长16%左右，社会消费品零售总额增长11%左右，城镇和农村牧区常住居民人均可支配收入分别增长9%和10%左右，城镇登记失业率控制在4%以内，居民消费价格涨幅控制在3%以内，完成自治区下达的节能减排任务。提出这些目标，综合考虑了可能与需要、当前与长远，总体上达到或高于自治区年度目标，既突出了加快发展、跳起来摘桃子的进取精神，又坚持从实际出发，体现了积极稳妥、切实可行的原则。

　　实现上述目标，决定性因素在于发展。我们要紧紧围绕发展这个第一要务，重点做好以下工作。

　　一、突出把握发展关键，全力推动项目投资实现较快增长

　　推进重点项目建设。扩大招商引资和项目建设冬季攻坚行动成果，建立重点项目建设清单，倒排工期，完成列入新开工计划项目的前期手续办理工作，确保施工季一到，集中开工100个以上重大项目。持续抓好项目策划，切实加强项目储备，实行重大项目动态管理、跟踪服务，增强项目建设和投资增长的连续性。全年实施投资5000万元以上重点项目700个以上，完成投资突破1200亿元。

　　加大招商引资力度。倾力打造良好投资环境，建立亲、清政商关系。瞄准京津冀、长三角、珠三角等重点地区，明确专人专班，有针对性地开展点对点招商，推进以商招商、产业链招商，千方百计落实项目、业主和投资，实现招商引资数量、质量的全面提升。全年签约落地投资亿元以上项目不少于100个，引进资金同比增长10%以上。

　　激发社会资本活力。认真落实中央、自治区关于支持非公经济发展、扩大民间投资的政策，制定我市具体配套措施，降低民间投资门槛，畅通民间投资渠道。大力扶持中小微企业发展，建立和完善配套服务体系，设立专项发展基金，着力解决融资难、融资贵等问题，推动中小微企业加强创新、改善经营、提高竞争力。在基础设施、公共服务等领域主推PPP模式，鼓励本土企业积极参与，年内落地PPP项目100个以上，全市PPP项目盘子超过2000亿元。

　　做大做实政府投融资平台。充分发挥各投融资集团的引领作用，在水利、矿业等领域整合组建新的国有资本投资公司，加强与金融机构和专业投资机构的合作，研究建立产业发展基金、股权投资基金，着力引进国内外私募股权投资基金，通过参股企业、持有债权等方式，更好地解决重点产业发展和重大项目融资问题。加快出台国资国企配套管理制度，明确经营方向、管理机制和投资决策机制，健全法人治理结构，加快提升资信等级，提高国有企业公司化、市场化、运营化管理水平。

　　二、持续积蓄发展动能，着力做大做强主导产业

　　千方百计做大做强矿产冶金和新型化工产业，挺起工业经济发展脊梁。推进云铜、金剑两个铜冶炼项目加快建设，确保到2018年形成百万吨优势产能。谋划铜业的全产业链发展，推动落地一批铜精深加工企业，形成产业集群，打造千亿元级产业。引进和培育有色金属产业基金，推动非标准有色金属交易中心尽快建成运营，促进铅锌银锡等矿产资源开发深度调整，以资本介入撬动产业水平提升。开工建设神雾集团70万吨乙二醇、大地云天二期60万吨磷肥、伊品生物基戊二胺等项目，推动大唐煤制气项目Ⅱ、Ⅲ系复工续建，确保大地云天一期60万吨磷肥、伊品生物10万吨苏氨酸和10万吨合成氨建成投产，推动形成化工产业的支柱地位。

　　千方百计培育新兴产业、改造传统产业，构建多极支撑的工业发展格局。发展新材料产业，加强硅产业领域的产学研合作，积极引入战略投资者，开工建设纳米级硅锆化工项目，同步规划精细化工园区，推进富勒烯产业园建设，打造特色产业集群。发展新能源产业，开工建设克旗120万千瓦抽水蓄能电站，力争全市新增电力装机110万千瓦。发展装备制造业，力促香港五龙集团新能源汽车、朝气集团航空装备制造产业基地项目开工建设。培育大数据产业，建成区域性大数据中心和智慧城市。加快发展生物医药产业，制定支持蒙中医药产业发展的政策措施，启动投资15亿元的泰领医疗海创生物科技园建设。改造提升食品、建材、纺织等传统产业，加大科技投入和产品创新力度，推动现有企业整合重组、做大做强，实现传统产业新一轮振兴。

　　千方百计推进3661工程，深化农牧业供给侧结构性改革。制定更加积极的扶持政策，发展设施农业、人工种草、高效畜牧业、丰产经济林和特色种养业，加快土地流转、规模经营和新型农牧业经营主体培育，确保完成年度3661工程任务。支持农牧业标准化生产，整合农畜产品品牌，集中力量打造体现地域特色的绿色有机产品。推进农牧业与二三产业融合发展，围绕建设绿色农畜产品生产加工基地，引进和培育有影响力的农畜产品加工领军企业，打造农牧业全产业链。推动电商下乡，加强农畜产品市场体系和快递服务网络建设，建成一批冷链物流和配送中心，以流通变革推动农村牧区经济转型升级。依托农牧业资源，积极发展旅游观光、休闲度假、养生养老、农耕体验等产业，拓宽农牧民就业增收渠道。搞好第三次全国农业普查，办好中国北方农博会。

　　千方百计推动文化旅游业实现新突破，促进现代服务业提档升级。围绕建设中国旅游强市和世界旅游目的地的目标，全力实施三步走战略，打一场旅游业跨越发展的翻身仗。集中精力推进一批王牌景区和核心景点建设，年内确保阿斯哈图石林景区晋升国家5A级景区，规划重塑一批新景区景点，开工建设以契丹辽文化博物馆为龙头的博物馆群，启动八里罕温泉小镇、美林谷冰雪小镇等30个特色旅游小镇建设。系统完成自驾游配套服务体系建设规划，全面启动27个自驾车营地和100个旅游咨询服务中心建设，加快建设旅游厕所、旅游标识系统、特色美食街区和旅游购物场所等配套服务设施，开工建设新能源汽车分时租赁一体化运营项目，力争旅游大厦建成竣工。实施旅游专线公路等级化两年攻坚行动，投资57.6亿元，全面开工建设53条、1000公里的重点景区连接线。推动文化、体育、商务等与旅游产业融合发展，出台鼓励民间创办博物馆的支持政策，策划、承办一批有影响力的会议会展、节庆活动、实景演艺和体育赛事，增加文化旅游产品的有效供给。围绕打造全家去赤峰、愉快又轻松旅游品牌，加大旅游宣传促销力度。成立市旅游发展委员会，设立旅游发展基金和专项引导资金。全年力争完成旅游投资250亿元，接待游客突破1600万人次，实现旅游收入260亿元。围绕建设区域性物流枢纽城市和商贸中心，健全现代商贸流通体系，以大流通促进商贸业大发展大繁荣。推动实体零售商业创新转型、线上线下互动，打造一批名店、名标、名牌。加快同方电子商务产业园建设，争创国家跨境电子商务试验区。加快发展智慧物流产业，抓好赤峰商贸物流城、金融物流港、联想冷链物流等项目建设，提高产城融合度。

　　千方百计培育激活金融产业，助推实体经济良性发展。全面落实已出台的支持金融业发展的一揽子政策，努力打通资本向实体经济顺畅流动的通道，切实在支撑实体经济发展的过程中推动金融业全面提升。对接国家绿色金融投资政策，积极引进外埠金融机构，鼓励驻赤金融机构设立分支，加快组建赤峰商业银行和赤峰金融控股集团。推动金融机构扩大信贷投放规模，力争新增贷款100亿元以上，贷款余额突破1500亿元。用好用足国家对贫困旗县IPO扶持政策，启动企业上市四年行动计划，推动内蒙古股权交易中心赤峰分中心尽快落地运营，指导和帮助企业拓宽直接融资渠道。推动保险业健康发展，探索保险资金服务实体经济的新模式新路径。积极搭建有效管用的银政企对接平台，创新市旗两级融资担保合作机制，做大做强政府融资担保业务。大力推广助保贷模式，年内放贷规模力争达到10亿元。

　　三、加快夯实发展基础，集中补齐城乡基础建设短板

　　进一步改善交通基础条件。加快立体交通建设，通过时空变革创造经济发展新优势。公路建设，积极争取规划支持，推进新惠至天山、灯笼河子至大兴一级公路建设，构建我市公路新的一纵一横主骨架。加快以国省干线为重点的高等级公路建设步伐，推动大板至经棚高速公路等8条在建公路早日建成，力促经棚至乌兰布统高速公路、赤峰至围场高速公路、小城子至平泉一级公路、天山至宝日勿苏一级公路等工程开工建设。年内力争完成公路建设投资100亿元以上。铁路建设，推进赤峰高铁等4个在建铁路项目，确保集通铁路增二线蒙大段全线开工，年内力争完成铁路建设投资40亿元以上。民航建设，启动并完成玉龙机场改扩建前期工作，力争林西支线机场具备开工条件，全面启动10个通用机场建设，确保阿旗通用机场实现首飞。

　　提升水资源保障能力。开工建设东台子水库和琥珀沟水库，确保三座店水库向中心城区引供水工程建成，力争跨区域调水工程具备开工条件。加快哈日朝鲁水库、沙那水库和47座大中型病险水闸除险加固工程建设，实施4个中型灌区续建配套与节水改造工程。完成中心城区供水安全保障规划，研究论证水源地迁出方案，并创造条件尽快组织实施。严格规范自备水源井管理和使用，启动中心城区关井压采工作，实施环中心城区农业节水试验工程和再生水回用工程，确保水资源综合利用水平得到较大提高。

　　加快能源通道建设。推动赤峰至华北超高压外送通道建设前期工作取得实质性进展。推进总投资5.2亿元的文钟220千伏输变电等6个电力工程建设，年内实现康家营、白音诺尔等电力工程竣工投运。改造升级157个中心村电网，完成4902眼机井配电工程，实现全市中心村电网升级和机井配电全覆盖。推进气化赤峰建设，完成赤峰至朝阳180公里输气管道工程，规划建设向翁牛特旗、宁城县、敖汉旗和喀喇沁旗城关镇的输气管线工程。

　　加强城市规划建设管理。科学统筹规划编制工作，实现总规、详规、专项规划有序衔接，切实强化规划执行。加快城市基础设施建设，实施总投资260亿元的重点市政工程。推进中心城区中环快速路、轨道交通、高铁赤峰西站综合交通枢纽、综合管廊、环城水系治理等项目加快建设。深入实施绿亮美和三山两园景观提升工程，打造绿色、休闲的宜居环境，全力创建国家园林城市。加大城关镇和重点小城镇市政基础设施和公共服务投入力度，提高城镇综合承载能力。制定加强城市管理的综合性措施。推进交通畅通工程，加大公共交通投入，改善居民出行条件。启动中心城区停车供给提升工程，开工建设红山区智能停车系统等项目，全面实施《中心城区车辆停放管理办法》，有效解决停车难、停车乱问题。切实加大融资力度，力争完成总投资500亿元、5.12万户的棚户区改造任务，改造老旧居民小区150万平方米以上。启动赤峰监狱搬迁工作。

　　加强园区基础设施建设。举全市之力建设赤峰城区东部工业经济走廊，完善管理体制机制，加大基础设施配套和公共服务投入力度，提高综合承载能力，推动国家高新区早日获批。开工建设园区自备电厂，全面启动马林有色金属产业园区道路和污水处理、固废处置、综合服务区等配套设施建设，年内完成基础设施投资30亿元以上，确保园区基础保障能力基本形成。充分挖掘比较优势，出台飞地经济配套政策，放大政策综合效应，引导各地实现重大项目有序流动、飞地布局。编制完成全市工业园区产业发展规划，明确各旗县园区的产业定位。设立园区发展基金，鼓励各地加大园区基础设施投入，切实打造实体经济集约集聚发展平台。

　　加强农村牧区基础设施建设。以美丽乡村建设为抓手，巩固农村牧区公共服务设施和村庄绿化成果，加大环境整治力度，建立投入和管护的长效机制，引导农牧民转变生产生活方式，持续改善农村牧区发展条件。选取中心城区及旗县城关镇周边有条件的村庄，开展集中供暖、供气、给排水和污水及垃圾综合处理试点，探索农牧民就地城镇化的有效途径。

　　四、着力创新发展路径，全面深化改革、扩大开放

　　推进供给侧结构性改革。严格落实国家和自治区去产能要求，年内淘汰煤炭产能210万吨。研究制定非商品住宅去库存政策，综合利用土地、税收等政策手段，推动房地产业稳定健康发展。加强政府债务管理，积极争取地方债券置换额度。合理利用国家投融资政策，加强金融监管和信用体系建设，确保不发生区域性、系统性金融风险。扩大大用户直供电额度，推动电力市场多边交易，争取启动增量配售电改革，降低工业企业用电成本；通过归并五险一金、实行税费减免缓和融资担保贴息等措施降低市场主体综合成本。加大政府投入力度，努力补齐教育、卫生、文化、社保等方面的突出短板，推动城乡基本公共服务均等化。

　　推进重点领域改革。深化放管服改革，以权责清单为抓手，全面推行双随机、一公开监管改革，完成公共资源交易平台整合工作，实现审批事项标准化、远程化管理。以城市管理、食药管理和旅游管理为突破口，全面推行综合行政执法。完成旗县区土地确权登记颁证和草原确权承包工作，探索开展土地所有权、承包权、经营权分置并行和宅基地入市交易、贷款抵押试点工作。完成国有林场改革，抓好集体林业综合改革试验示范工作。深化水务一体化改革，成立赤峰市水资源管理与保护委员会，推进水权交易，启动水价调整。深化供销合作社综合改革，打造服务农牧民生产生活的综合平台。在红山区试点开展城镇社区信息化改革，并逐步在全市推开。推进行政嘎查村社区化管理体制改革，完善提升农村牧区便民服务中心功能。推进足球改革试点工作。完成盐业管理体制改革。

　　全方位扩大开放。围绕做好融入京津、拓展东北、向北开放、转身向海四篇文章，不断提高开放型经济发展水平。深度融入京津冀、东三省、环渤海区域发展，努力打通基础设施、产业合作、人文交流的渠道。密切与北京的对口合作关系，打造自治区融入京津冀协同发展的先行区。推进北向锡赤铁路通道的连通和延伸，加强与环渤海港口的合作联系，大力发展出口加工贸易。开发拓展赤峰保税物流中心功能，创造条件申建国家综合保税区。积极承接发达地区产业转移，争创国家级产业合作升级示范区。积极参与一带一路建设，承办好锡赤通三盟市与蒙古国三省区域合作会议，构建外向型经济发展新格局。

　　五、注重共享发展成果，切实保障和改善民生

　　狠抓脱贫攻坚头号民生工程。加大产业扶贫力度，通过培育特色产业、实施增收项目、鼓励农牧业新型经营主体参与等方式帮扶贫困户脱贫。推广产业园区+易地扶贫搬迁模式，年内完成1.3万建档立卡贫困人口搬迁任务。大力推进金融扶贫，推动强龙贷放款规模翻一番、总规模50亿元的扶贫产业基金落地放款。推广敖汉旗健康扶贫做法，设立贫困人口大病保障基金，构建政府、市场、社会协作的健康扶贫工作格局，实现贫困人口签约服务和免费健康体检全覆盖。建立扶贫攻坚1+N配套政策体系，向国贫旗县派驻脱贫攻坚推进组、向区贫旗区派驻脱贫攻坚督导组，强化驻村帮扶工作，实现贫困村驻村工作队全覆盖。加大督查巡查、考核评估力度，实行挂图作战、动态管理，引入第三方评估机制，严格落实各方责任，加快实现精准脱贫。年内确保超额完成5.8万贫困人口稳定脱贫任务，实现松山区、克旗两个区贫县摘帽。

　　推进就业创业工程。发挥赤峰安快创业谷引领示范作用，推动旗县区普遍建设众创空间，实现以创业带动就业。健全城乡均等的公共就业服务体系，统筹做好高校毕业生等重点群体的就业工作，加大政府购买基层公共管理和社会服务岗位力度，年内安置就业困难人员4000人，新增城镇就业3.15万人，农牧民转移就业稳定在68万人，城镇零就业家庭动态清零。推进就业培训体制改革，加大农牧民培训力度，完成职业技能培训3.5万人次以上。

　　提高社会保障水平。持续加强社会保障体系建设，推动社会保险制度改革，实施全民参保登记计划，提高优抚对象抚恤金和城乡低保补助金标准，推进社会保障由制度全覆盖向人员全覆盖转变。支持社会慈善、社会捐助、社会福利等事业发展，完善各类救助体系建设，做好留守儿童、孤儿、残疾人、流浪乞讨人员等弱势群体的关爱、保障工作，筑牢困难群体基本生活保障网。

　　促进各类教育统筹发展。加快建设公办幼儿园，大力发展普惠性幼儿园，扩大学前教育资源供给。推动义务教育均衡发展，力争5个旗县区通过国家评估认定。全面实施普通高中优质特色多样化学校建设，推动全市基础教育跨越升级。深化自治区职业教育综合改革试验区建设，推进赤峰职教园区建设，加快构建现代职业教育体系。推进赤峰学院应用型本科转型发展，提升服务地方经济社会发展的能力。优先重点发展民族教育，全力实施好民族学校标准化建设工程。加强教师队伍建设，实施卓越校长、品牌学校行动，推进城乡校长教师轮岗交流，建设教育云平台，增强教育事业发展保障能力。

　　加快健康赤峰建设。做好重大疾病防控工作，启动城市公立医院综合改革，全面推行分级诊疗制度。加强与国药集团、阳光保险等实力企业的合作，多渠道筹措建设资金，实施市医院扩建工程，新建市中蒙医院、传染病医院、中心血站，加快妇儿医院建设重组，以市场化的办法力促康复医院和血透、消杀、影像、医疗器械维修、临床检验五个中心开工建设，进一步丰富和完善市级医疗供给。促进两孩政策和优生优育措施全面落实。开展丰富多彩的全民健身活动，实现全民健身活动中心县城全覆盖。加快发展养老产业，推广医养结合模式，推动形成以居家为基础、社区为依托、机构为支撑的养老服务体系。

　　各位代表，为了展示党的民族区域自治政策的伟大成就，为了回应社会关切、解决群众关注的热点难点问题，我们整合统筹市级主导的各类民生项目，系统提出总投资200亿元的迎庆自治区成立70周年十大民生工程，通过工程献礼促进项目实施，集中推动民生工作跨越提升。具体包括：中小学、幼儿园办学条件提升工程，宽带进乡村工程，行政嘎查村全面通油路工程，气化赤峰天然气长输管道工程，市级医疗机构布局调整及改造提升工程，市级公共服务设施补短板工程，中心城区市政基础设施补短板工程，中心城区公共交通提档升级工程，中心城区饮水安全保障工程，公共文化重点建设工程。上半年确保26个具体项目全面开工，年内基本完成大部分项目建设。

　　六、切实强化发展保障，努力维护公共安全和社会稳定

　　坚决扭转安全生产工作被动局面。深刻吸取12·3事故的沉痛教训，启动安全生产监管执法年活动，认真落实管行业必须管安全、管业务必须管安全、管生产经营必须管安全的系列要求，持续开展安全生产大检查，坚决避免重特大安全生产事故发生。切实压实企业的安全生产主体责任，依法从严处理违法违规的生产经营行为。严格落实党政同责、一岗双责，坚决实行一案双查和最严厉的追责问责。厘清行业部门安全生产监管范围，建立监管责任清单，依单履责、失职追责、尽职免责。创新安全生产监管方式，设立有奖举报基金，加大宣传教育培训及曝光力度，在重点行业和重点地区建立数字化安全监控系统和社会化专业化服务体系。加强市县安全监管力量，做实各类园区和苏木乡镇的安监机构。建立安全生产防范预警机制，完善事故应急预案，加强应急救援队伍建设，提高事故应急处置和救援能力。

　　加强市场安全监管。加大市场监管的基层基础建设力度，推动监管力量下沉，消除监管盲区，确保群众消费安全。加强农作物病虫害防治、畜禽疫病防控和农畜产品质量监管，加快建立从农田牧场到餐桌的全过程可追溯管理体系，确保农畜产品质量安全和产业发展安全。立足集中规范监管，确保每个旗县区建成一处以上食品小作坊集中加工园区和标准化农贸市场。启动市综合检验检测中心建设，推行全行业信息化监管，研究建设全市食品药品安全云平台，加快实现餐饮单位信息化管理全覆盖。

　　抓好环境保护工作。继续实施好大气、水、土壤污染防治工作计划，下力气提升河流水质，严控重金属污染，启动废弃厂矿填埋工作，保持打击环境违法行为高压态势，不断改进环境整体质量。严格项目准入环保门槛，着力推进节能减排工作，全面取缔10蒸吨以下燃煤锅炉，完成自治区下达的黄标车淘汰任务。持续推进自然资源资产负债表编制，建立健全环境保护治理的常态长效机制。推行环境污染第三方治理，加快排污权市场化改革。

　　推进生态建设。加快实施京津风沙源治理、天然林保护、退耕还林还草等重点生态工程，力推高效丰产经济林、樟子松、文冠果、山杏林、沙化土地综合治理五大基地建设，全年完成林业生态建设100万亩、水土保持治理150万亩。巩固重点区域绿化成果，进一步加大工作力度，完成高等级公路两侧绿化工程，推进环中心城市生态经济圈建设，年内全市完成重点区域绿化20万亩。认真落实禁牧休牧和草畜平衡条例、草原生态保护补助奖励政策，以法制手段大力强化生态保护，持续改善生态环境。

　　保持社会大局安全稳定。深入推进平安赤峰建设，强化重大决策风险评估，完善立体化社会治安防控体系，完成中心城区公共安全视频监控系统建设，提升风险防控和公共安全水平。加强信访工作，健全大调解工作格局，努力畅通群众诉求表达、利益协调、权益保障渠道，有效规范信访秩序。创新社会治理，提高预知预警能力，严厉打击各类违法犯罪活动，坚决防止个人极端事件和危害公共安全事件发生，不断提升人民群众安全感和满意度。

　　七、积极营造发展环境，不断加强民主法治和精神文明建设

　　坚持推进依法治市。科学编制立法工作规划，推动生态环保、城市管理、水资源保护等重点领域立法。推进依法行政，促进规范公正文明执法，建设职能科学、权责法定、执法严明、守法诚信的行政执法和监督体系。支持检察机关公益诉讼，深化行政执法与刑事司法衔接机制建设。深入实施七五普法，提高全社会法治意识。

　　全面接受各方监督。主动接受人大及其常委会的法律监督和工作监督，认真执行人大各项决议，定期向人大报告工作，做好代表意见办理工作，提高政府决策的科学化、民主化、法制化水平。自觉接受政协的民主监督，实施好政府工作协商计划，广泛听取各民主党派、无党派人士的意见建议，认真办理委员提案。全面接受社会监督，广泛听取人民群众的呼声，以社会舆论为风向标，主动改进政府工作。

　　加强精神文明建设。以创建全国文明城市为载体，在全市大力弘扬六德文化，建设德善赤峰。充分发挥乡村文化站所作用，组织实施全市农牧民文艺汇演，不断丰富群众文化体育生活。加强精品文艺创作，办好蒙冀辽文化产业博览会和全区乌兰牧骑艺术节，承办好中华一家亲那达慕大会，加快辽文化和红山文化遗址申遗进程。完善社会信用体系，建设诚信赤峰。大力弘扬良好的家风、村风、行风、政风，通过新风正气制度化、常态化，夯实社会和谐的基础。

　　团结凝聚各方面力量。注重发挥老干部、工商联、工会、共青团、妇联、红十字、科协、文联等组织的桥梁纽带作用，充分调动中区直驻赤单位的积极性、主动性，凝聚广泛共识，形成建设现代化赤峰的强大合力。发扬双拥共建的优良传统，转变人民防空发展方式，促进军民融合发展。认真贯彻民族工作方针政策，深入开展民族团结进步教育和创建活动。依法管理宗教事务，发挥好宗教团体、宗教界人士和信教群众在促进经济社会发展中的积极作用。

　　八、不断提升引领发展能力，进一步加强政府自身建设

　　坚持不懈强化治庸治懒、忠诚担当。增强政治意识、大局意识、核心意识和看齐意识，深刻领会、准确把握、认真落实中央、自治区和市委的各项决策部署，使政府工作始终在正确的轨道上推进。建立健全两学一做常态化、制度化机制，按照市委要求，开展好比实干、比担当、比奉献解放思想主题教育实践活动。健全奖勤的办法，让认真履职、勇于创新者有为有位；加大罚懒的力度，让不想、不敢干事者受到惩戒；创新治庸的手段，让在其位不谋其政、浑浑噩噩者没有空间；建立容错的机制，让改革创新、敢于担当者放手放胆做事。

　　坚持不懈强化求真务实、真抓实干。把抓落实作为检验政府工作成效的核心标准，大力发扬马上就办的精神、钉钉子精神和蒙古马精神，提高政府执行力和行政效率。加强政府工作人员的教育培训，切实以习近平总书记系列重要讲话精神和治国理政新理念新思想新战略武装头脑、指导实践、推动工作。坚持一张蓝图绘到底，在重点工作和重大项目上用真劲、下真功、求实效，努力用干部的辛苦指数换取经济社会的发展指数和人民群众的幸福指数。加大督查工作力度，完善督查工作机制，确保政府各项工作件件有着落、事事有回音。

　　坚持不懈强化主动作为、服务大局。牢固树立大服务的理念，为广大群众、投资客商以及全社会搞好服务，以服务推动发展、引导投资、促进建设。创新政务服务，实施互联网+政务服务等信息惠民工程，加快政务云平台建设，力推网上审批、网上办事，让信息多跑路、群众少跑腿。推进政务公开，增强政府工作透明度。健全新闻发布会制度，及时回应社会关切。

　　坚持不懈强化干净做事、清正廉洁。认真落实一岗双责，深化廉政风险防控，更加自觉遵守中央八项规定、自治区28项配套规定和市委44项具体规定，坚决防止四风反弹回潮。强化行政监察和审计监督，严格规范公共权力行使和公共资源配置，及时发现、坚决纠正、严厉惩处违法违规违纪行为，努力打造廉洁从政的公务员队伍，树立风清气正的政府形象。

　　各位代表！新征程赋予新使命，新目标呼唤新攀登。让我们紧密地团结在以习近平同志为核心的党中央周围，在自治区党委和市委的坚强领导下，不忘初心、守望相助、一往无前，撸起袖子加油干，全力加快赤峰跨越崛起的步伐，以优异成绩迎接党的十九大胜利召开和自治区成立70周年，谱写好祖国北疆亮丽风景线的赤峰篇章！
